# Goulphar: rapid access and expertise for standard two-color microarray normalization methods

**DOI:** 10.1186/1471-2105-7-467

**Published:** 2006-10-23

**Authors:** Sophie Lemoine, Florence Combes, Nicolas Servant, Stéphane Le Crom

**Affiliations:** 1 IFR36, Plate-forme Transcriptome, École Normale Supérieure, 46 rue d'Ulm, 75230 Paris cedex05, France; 2 INSERM U784, École Normale Supérieure, 46 rue d'Ulm 75230 Paris cedex05, France

## Abstract

**Background:**

Raw data normalization is a critical step in microarray data analysis because it directly affects data interpretation. Most of the normalization methods currently used are included in the R/BioConductor packages but it is often difficult to identify the most appropriate method. Furthermore, the use of R commands for functions and graphics can introduce mistakes that are difficult to trace. We present here a script written in R that provides a flexible means of access to and monitoring of data normalization for two-color microarrays. This script combines the power of BioConductor and R analysis functions and reduces the amount of R programming required.

**Results:**

Goulphar was developed in and runs using the R language and environment. It combines and extends functions found in BioConductor packages (limma and marray) to correct for dye biases and spatial artifacts. Goulphar provides a wide range of optional and customizable filters for excluding incorrect signals during the pre-processing step. It displays informative output plots, enabling the user to monitor the normalization process, and helps adapt the normalization method appropriately to the data. All these analyses and graphical outputs are presented in a single PDF report.

**Conclusion:**

Goulphar provides simple, rapid access to the power of the R/BioConductor statistical analysis packages, with precise control and visualization of the results obtained. Complete documentation, examples and online forms for setting script parameters are available from .

## Background

DNA microarray technology has made it possible to monitor gene expression on a large scale. However, one of the major limitations of this technology is that biochips are subject to variations from the start of the experimental process, right to the end of the analysis. These variations depend on the experimental and technical biases inherent to the biological samples, dye incorporation methods and hybridization steps used [1-3]. Several normalization methods have been developed for correcting systematic biases, but there is currently no universal method that can be systematically applied [4]. Each method has its own specific features, such as correcting for dye biases by global lowess normalization or compensating for spatial artifacts using a print-tip median correction. It is not always obvious which normalization method is most appropriate. Many of these methods are implemented in the R programming language [5] and are part of the BioConductor project [6]. However, although effective methods exist, R is not easy to use for those not familiar with programming language, as is the case for most scientists using microarrays.

Goulphar combines and extends the two-color microarray normalization methods and basic graphics functions of various R/BioConductor packages, applies customizable filters to spot properties and provides information for monitoring the effects of the normalization method applied in the form of 1) images of the data before and after normalization, 2) spatial plots of the data and valid spot numbers per print tip, making it possible to adapt the normalization method to the bias present on the slide. Goulphar directly processes the scanner output files, thereby avoiding problems arising from the conversion or manipulation of such files [7].

## Implementation

The Goulphar script (additional file 1) runs under the R command environment. It works with the raw data file and a parameter file created by the user. The parameter file is a simple text file containing the values for each parameter. The online documentation describes the content and available values for each parameter. A sample parameter file is provided, as additional file 2. An online Internet form has been developed for selecting parameters and threshold values. The script can also be run on our server, but with fewer options than are available when the script is used on a local computer. Goulphar creates a graphical output, in a report file (see additional file 3), and outputs the normalized expression data in a tabulated text file.

## Program overview

### Data pre-processing and normalization

A pre-processing step is required before normalization, to eliminate artifactual signals and to correct for the background. Goulphar includes four optional data pre-processing steps: 1) subtraction of the background signal, 2) elimination of the spots flagged up by the image analysis software, 3) filtering out of saturated spots with an intensity exceeding a given threshold value, and 4) elimination of spots with a diameter smaller than a predefined value. All filtered spots are excluded from both normalization and graphical output.

Goulphar uses various two-color microarray normalization methods from the limma package [8] for the print-tip lowess, global lowess and global median methods and for calculation of the print-tip median. This script also extends the limma package including global lowess normalization followed by print-tip median correction.

### Output: diagnostic plots and normalized data

Goulphar was developed for the monitoring of normalization. We therefore focused on creating graphical output. Most of the plotting functions have been adapted and combine plots from the limma and marray BioConductor packages [9]. Goulphar generates MA plots and box plots before and after normalization, to evaluate the influence of the method used. These graphical outputs are created for each step of the normalization process. For the global lowess followed by print-tip median normalization method, graphical output is created before normalization, after the global lowess step and after the print-tip median correction. This output is essential for checking that each normalization step has been performed correctly.

For the print-tip lowess normalization method, an additional plot displays the number of unfiltered spots in each block (see additional file 4). This plot alerts the user when the number of spots in a given block is too low (the block number is shown on the graph). In such cases, the print-tip lowess normalization method must be used with care, as it may also take into account biological variation.

The script displays density distributions for both channel intensities and M values before and after normalization. Intensity plots are used to check that the dye bias encountered in two-color microarray analysis has been corrected properly. M value plots are useful for assessing the dispersion of results, as scattering must be minimal for most normalization methods.

Finally, Goulphar builds a suite of diagnostic plots. Two background intensity plots for both channels (Cy5 and Cy3), before and after data pre-processing, are used to control the hybridization and washing steps. These plots facilitate direct monitoring of the accuracy of the image analysis flagging process. A map of the filtered spots is built so that the user can localize the spots excluded from normalization. Maps representing M values on the array help detect spatial biases, such as washing-induced asymmetry. As for MA plot and box plot representations, these plots are generated before and after each normalization step. Finally, a map of mean signal intensity (A values) on the array is built. This map is used to detect intensity biases due to variations in probe concentrations between plates, for example.

All these plots can be included in a single report or saved as independent PNG or JPEG files. Goulphar also generates a tabulated text file containing the normalized ratio (M values) and information about spot intensities: A values, red and green raw intensities and background.

### Advantages of Goulphar

Goulphar was designed with the primary aim of providing easy access to powerful statistical analysis methods for microarray data based on the R and BioConductor packages. Goulphar is more versatile than the R-based tools available through online web servers, such as ArrayPipe [10], CARMAWeb [11], DNMAD [12], MIDAW [13], SNOMAD [14] or WebArray [15]. It runs independently of network access, overcoming the need for large file upload and management, and provides direct access to the source code, facilitating modifications and improvements not possible in other "closed" resources (ArrayPipe, DNMAD or WebArray). Goulphar combines functions and plots found in different BioConductor packages, whereas to limmaGUI [16], the graphical user interface for the limma package is restricted to limma.

The use of Goulphar as a graphical interface simplifies and standardizes access to BioConductor functions. All the parameters selected by the user are compiled into a single file and read by the Goulphar script using only one command line. This considerably simplifies the application of a given set of parameters to various files. There is no need to adapt the input file format (as in MIDAW or SNOMAD) or to go through multiple online forms (ArrayPipe and CARMAWeb). Working with a parameter file simplifies the storage and follow-up of data during and after normalization. This tracking is important if the MIAME microarray standard is to be respected [17].

Goulphar also includes improvements to the BioConductor packages in the pre-processing of scanner output files before normalization. First, the filtering of artifactual spots can be customized, whereas this function is not fully implemented in other packages. Second, the user can choose whether to subtract the background, an issue still much debated in the microarray community [18]. The spot filtering parameters used after flagged image analysis has been detailed, making it possible to filter only spots discarded by the user, retaining the other spots flagged by the software, when using GenePix Pro image analysis software (Axon Instruments, Foster City, CA, USA), for example. Weak spots and spots with no signal are often automatically discarded but are useful for normalizing ratios for the lowest intensities by the lowess method. Other programs lack methods for handling artifactual spots (MIDAW and SNOMAD) or are restricted to the discarding or retaining of all flags (DNMAD, CARMAWeb and WebArray).

Goulphar also extends the normalization methods found in limma by combining global lowess and print-tip median corrections. The global lowess normalization is carried out first and takes dye biases into account. This method is applied to all the spots, giving the highest degree of accuracy and fewer modifications of the relevant signal. The print-tip median method then corrects for spatial artifacts, with no limitation on the number of spots used per block. Other programs allow a choice only between global or local methods, and the print-tip lowess correction is applied with no control over the number of spots used for the calculation (ArrayPipe, CARMAWeb, DNMAD, MIDAW and WebArray). SNOMAD combines global and local normalization methods but performs only a global median correction, without lowess correction. Our two-step correction results in the accurate normalization of systematic biases whilst avoiding most of the limitations of each method applied separately.

R can be used to generate graphics, but it is not straightforward to set all the parameters required to obtain high-quality figures. In the development of the Goulphar script, considerable effort went into grouping all the graphical output and parameters used into a single PDF file, so that the user only need consult this report and the normalized results. The CARMAWeb web service is the only other tool to offer this possibility. PDF reports are very useful in the daily management of core microrarray facilities involved in data analysis.

The way in which Goulphar has been developed makes it easier to implement new functions. For example, the script was initially designed to work with GenePix image analysis software, but has also been adapted to the Spot image analysis system (CSIRO, Clayton South, Australia). Goulphar is flexible, as it is can be adapted to deal with a new type of input data and new normalization methods can be introduced directly in source code.

## Conclusion

Goulphar is versatile and very easy to use, with a single command line and extensive graphical output presented in a single PDF file, making it popular among users. Goulphar combines functions and plots from BioConductor packages and extends them with customizable filtering options and a larger number of plots alerting the user to particular problems. Goulphar is a flexible solution, as it can be used alone with the R environment or integrated into a more complex workflow. Finally, Goulphar is also a good starting point for experimental scientists without extensive programming skills who want to make use of the powerful R packages on their computer.

## Availability and requirements

**Project name: **Goulphar

**Project home page: **

**Operating system(s): **Multiplatform, as it uses the R software available in the Windows, MacOS and Linux environments [5].

**Programming language: **R

**Other requirements: **R software and the following packages: marray, limma, convert and hexbin

**License: **The R script is available and distributed under the GNU General Public License [19].

## Authors' contributions

SL programmed most of the R code improvements in the Goulphar script, writing new combined normalization functions and creating the web pages and the online form. FC worked on the PDF report output. NS developed the first implementation of the script. SLC initiated and coordinated this project. SLC was also involved in script testing and improvements. All authors read and approved the final manuscript.

## Supplementary Material

Additional file 1 **The Goulphar R script**. This is the Goulphar script code in R programming language. This text file runs in the R environment and can be read and modified in a text editor. The script needs a parameter file (see additional file 2) and a raw data file (see additional file 6), located in the same folder, to work properly. Click here for file

Additional file 2 **Example of a Goulphar parameter file**. Here is an example of a parameter file used by the Goulphar script. This is a tabulated text file that can be opened and manually edited using a text editor. The parameter file available here was set up with the following options. The median has been selected for the foreground, the flags from the image analysis software have been filtered out using -50, -75 and -100 values (from the GenePix Pro image analysis software), no background subtraction has been done, saturating spots with intensities over 60,000 in one of the two channels have been filtered out, and no filter was applied to spot diameter. The normalization method used was global lowess followed by print-tip median local correction. The output plots are presented in a single PDF report file (see additional file 3). A complete list of all the available parameter options can be found on the online help page of the web site or in the PDF documentation provided as additional file 5. Click here for file

Additional file 3 **Example PDF report output created by the Goulphar script**. The PDF file provided here is typical of the output obtained from the Goulphar script. This report was obtained after launching Goulphar on the image analysis output from additional file 6, using the parameter file from additional file 2. Click here for file

Additional file 4 **Print-tip lowess quality control plot**. This plot is obtained when the "print-tip group lowess" normalization method is selected. It displays the number of spots, in each print-tip group, kept for normalization once the filtration process has been performed (i.e. removing artifactual spots). The lowess correction method is sensitive to the number of spots used for the calculation, and this method is not appropriate if the there are too few spots in one block. Blocks with too few spots are displayed on the graph (here blocks 21, 25 and 29) to help the user to assess the efficiency of local lowess normalization. The maximum number of spots to be expected in each block, from the slide layout analysis, is displayed below the x-axis label. The experiment on which this plot is based was performed on yeast microarrays (Véronique Tanty personal communication). Click here for file

Additional file 5 **Goulphar script documentation**. The documentation of the Goulphar script deals with all the options available for parameter file creation and all the outputs that can be obtained. Click here for file

Additional file 6 **A sample microarray result file**. The microarray result shown here was obtained with GenePix Pro 6.0 image analysis software (Axon Instruments, Foster City, CA, USA). This file was obtained by analyzing a yeast whole-genome microarray to identify *YRR1 *transcription factor targets [20]. This raw data file was used for all the examples of the script interface and outputs shown (additional file 2 and additional file 3). Click here for file
