# Supplementary material for: Goulphar: rapid access and expertise for standard two-color microarray normalization methods
Source: BMC Bioinformatics. 2006 Oct 23;7:467. doi: 10.1186/1471-2105-7-467 (PMC1626094; doi:10.1186/1471-2105-7-467)
Supplement: Additional file 3 — Example PDF report output created by the Goulphar script. The PDF file provided here is typical of the output obtained from the Goulphar script. This report was obtained after launching Goulphar on the image analysis output from additional file 6, using the parameter file from additional file 2. [file 1471-2105-7-467-S3.pdf]

Normalisation report for yeast_slide.gpr

GPR file = yeast_slide.gpr

Foreground = median foreground

Flags = remove −50 −75 −100

Background = no background subtraction

Saturating spot filter = saturating spot threshold = 60000

Spot diameter filter= No spot diameter filter

Normalisation type = global lowess followed by a block median normalisation

Graphical output= in pdf report

Red (Cy5) background intensity including filtered spots

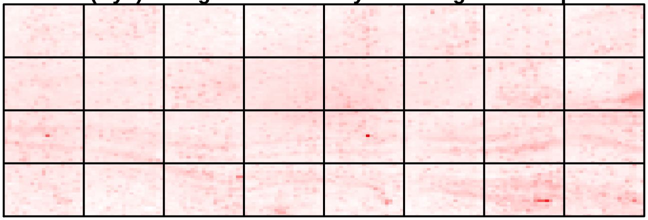

Red (Cy5) background intensity excluding filtered spots

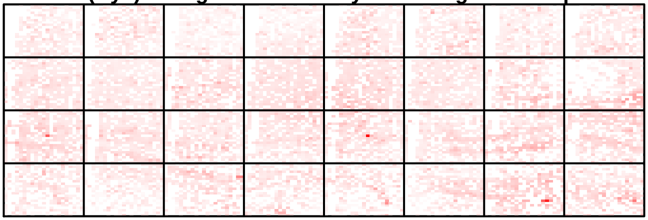

Green (Cy3) background intensity including filtered spots

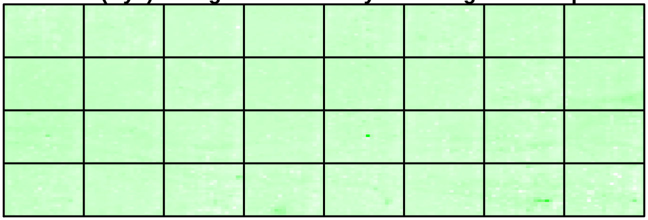

Green (Cy3) background intensity excluding filtered spots

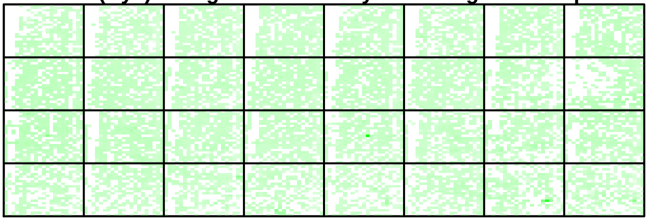

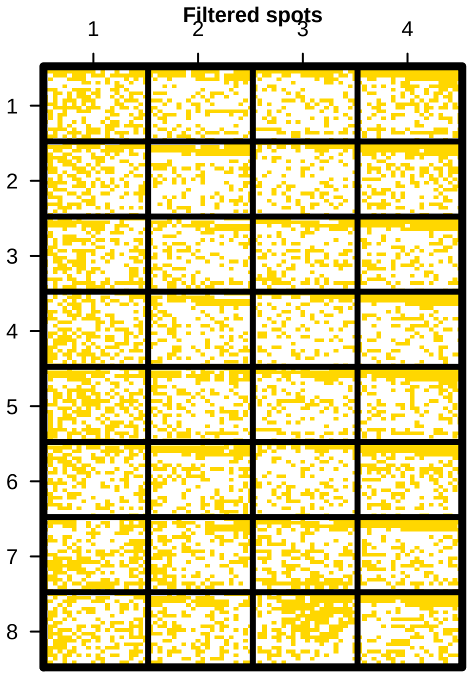

Filtered spots
1
2
3
4
1
2
3
4
5
6
7
8

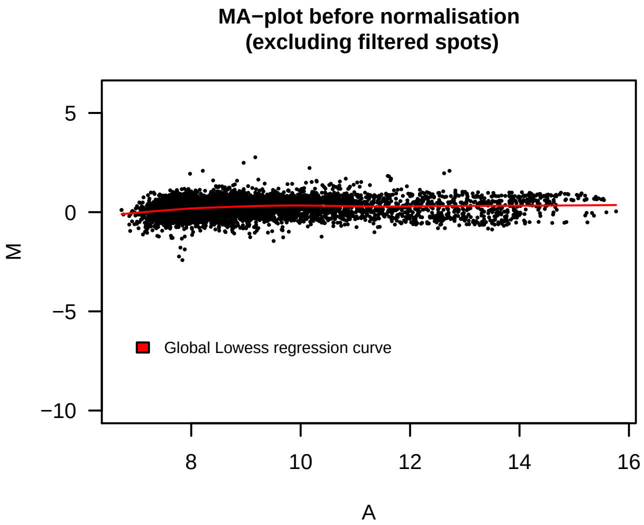
MA−plot before normalisation
(excluding filtered spots)
5
0
−5
−10
M
8
10
12
14
16
A
Global Lowess regression curve

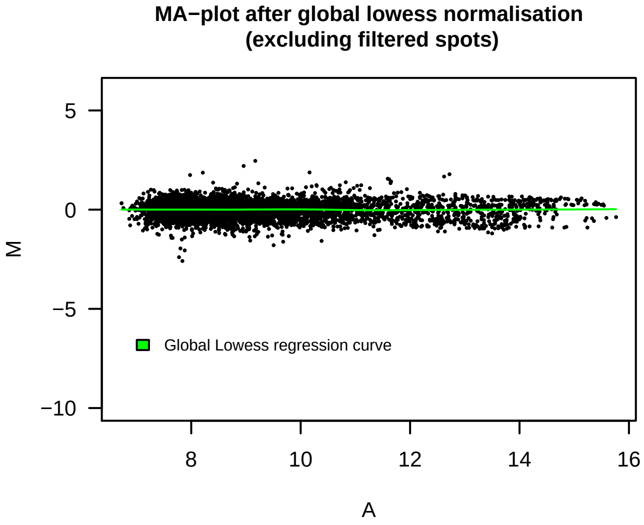
MA−plot after global lowess normalisation
(excluding filtered spots)
5
0
−5
−10
M
8
10
12
14
16
A
Global Lowess regression curve

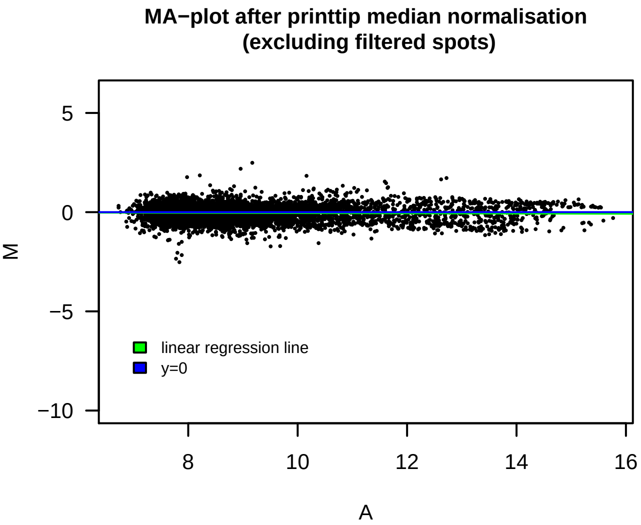

MA−plot after printtip median normalisation
(excluding filtered spots)
5
0
−5
−10
M
8
10
12
14
16
A
linear regression line
y=0

Boxplot of printtip groups before normalisation (excluding filtered spots)

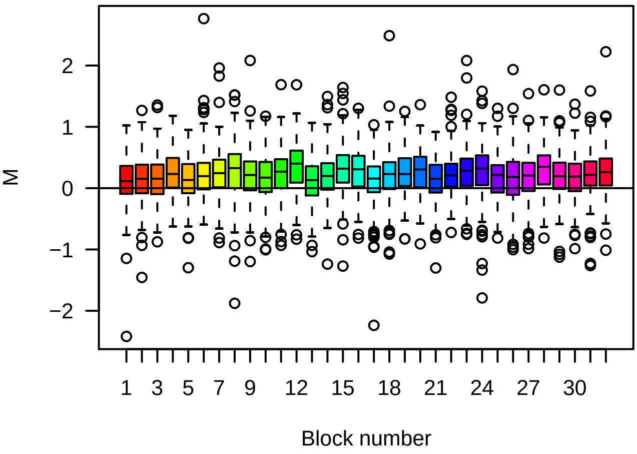

Boxplot of printtip group after global lowess normalisation (excluding filtered spots)

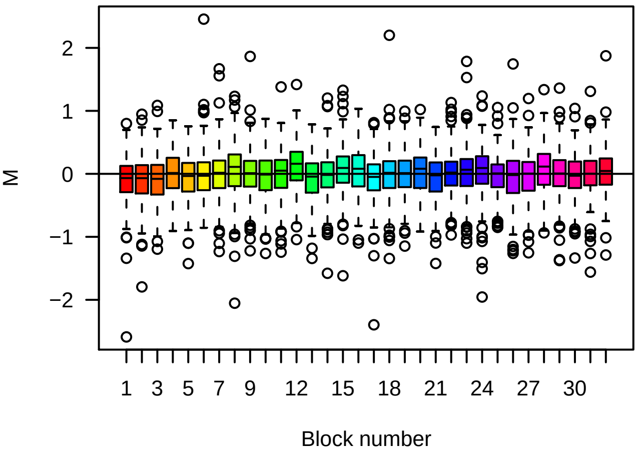

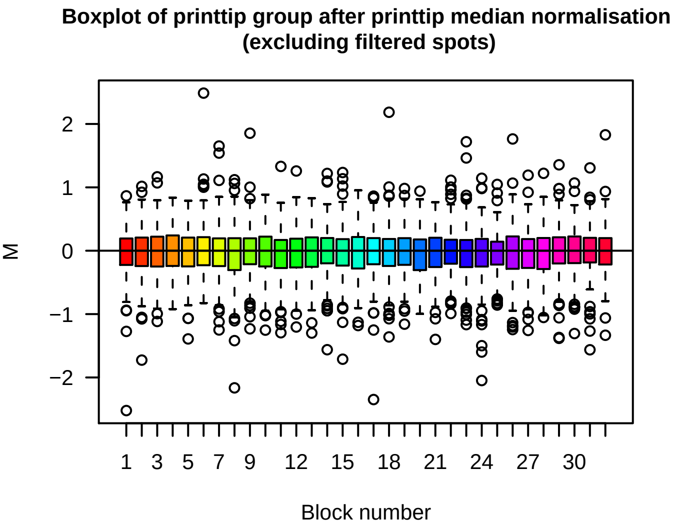
Boxplot of printtip group after printtip median normalisation
(excluding filtered spots)
M
Block number
2
1
0
−1
−2
1
3
5
7
9
12
15
18
21
24
27
30

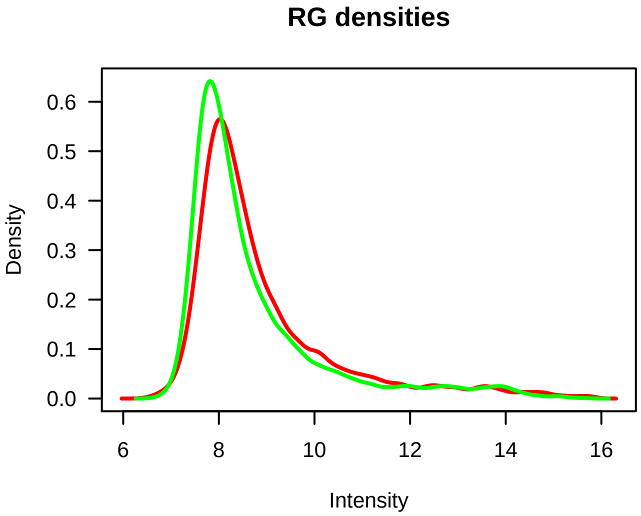
RG densities
Density
0.0
0.1
0.2
0.3
0.4
0.5
0.6
6
8
10
12
14
16
Intensity

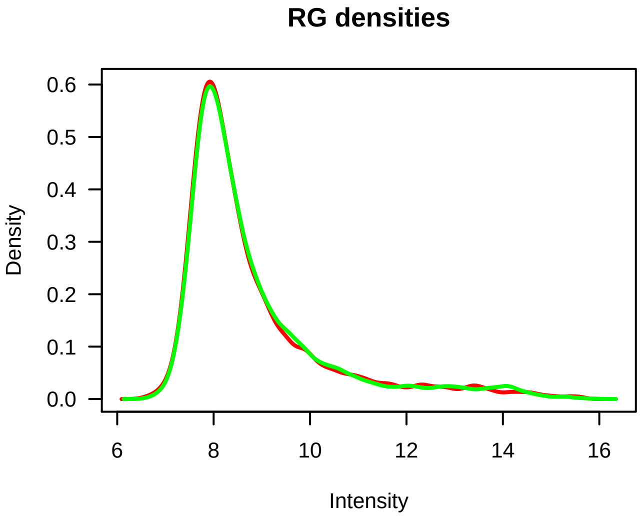
RG densities
Density
0.0
0.1
0.2
0.3
0.4
0.5
0.6
6
8
10
12
14
16
Intensity

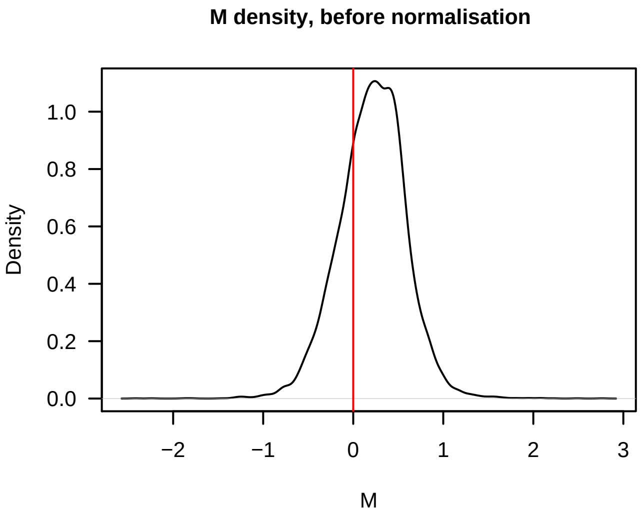
M density, before normalisation
Density
1.0
0.8
0.6
0.4
0.2
0.0
−2
−1
0
1
2
3
M

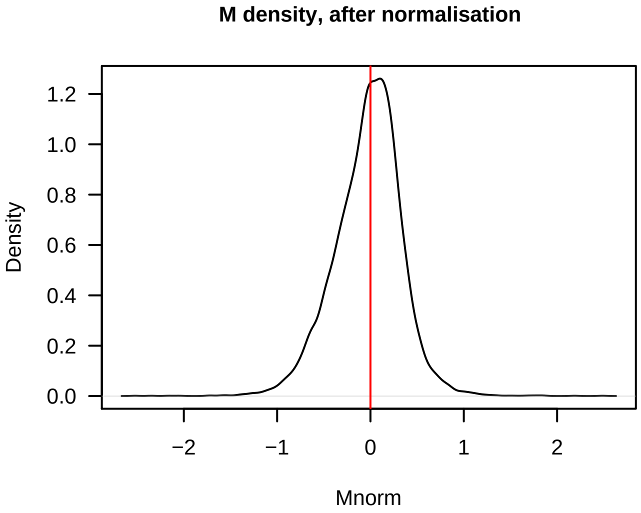
M density, after normalisation
Density
1.2
1.0
0.8
0.6
0.4
0.2
0.0
−2
−1
0
1
2
Mnorm

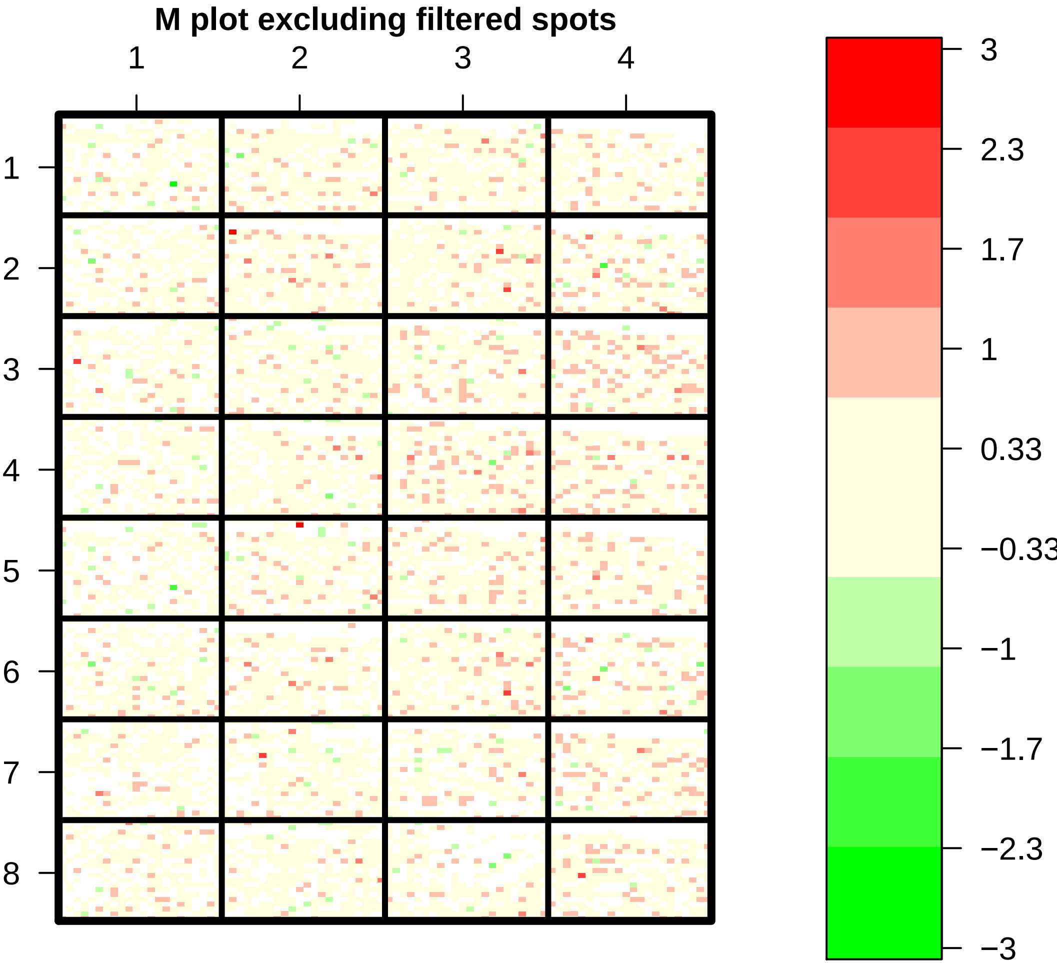

M plot excluding filtered spots
1
2
3
4
1
2
3
4
5
6
7
8
3
2.3
1.7
1
0.33
−0.33
−1
−1.7
−2.3
−3

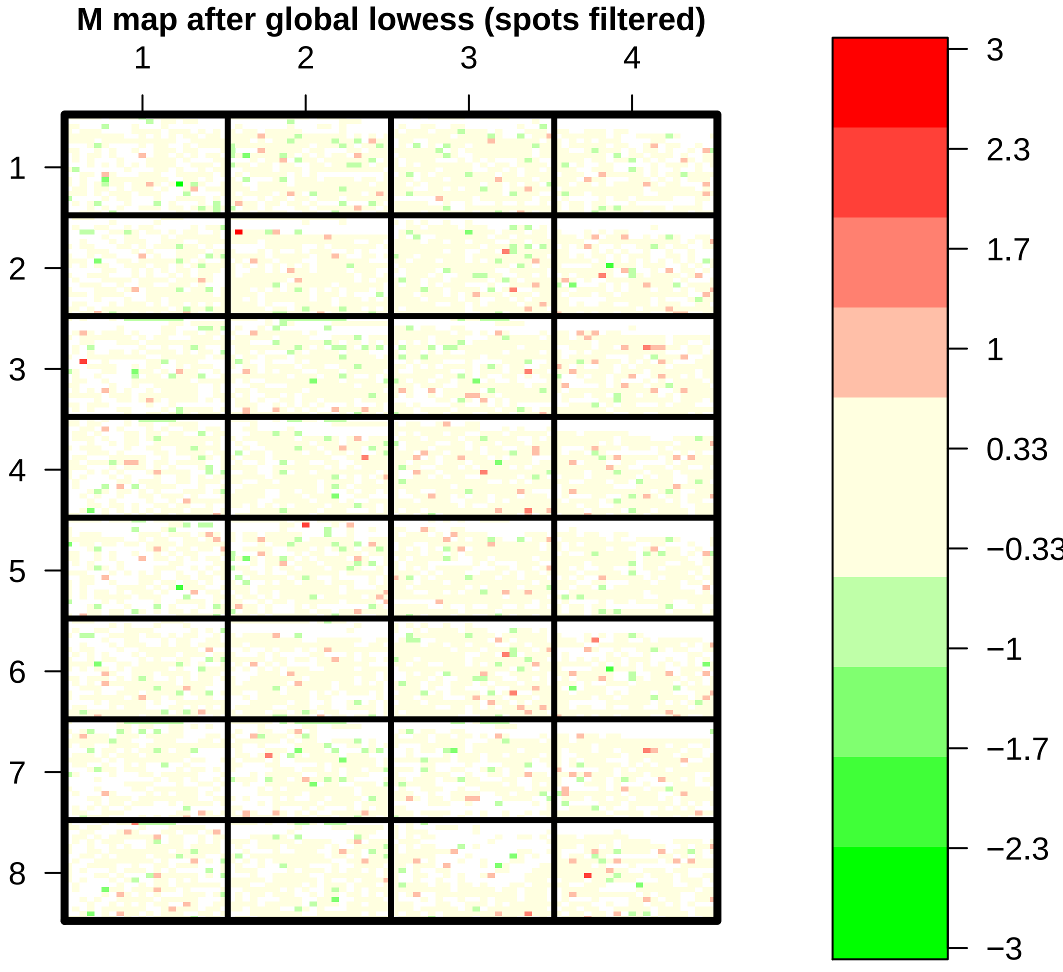
M map after global lowess (spots filtered)
1
2
3
4
1
2
3
4
5
6
7
8
3
2.3
1.7
1
0.33
−0.33
−1
−1.7
−2.3
−3

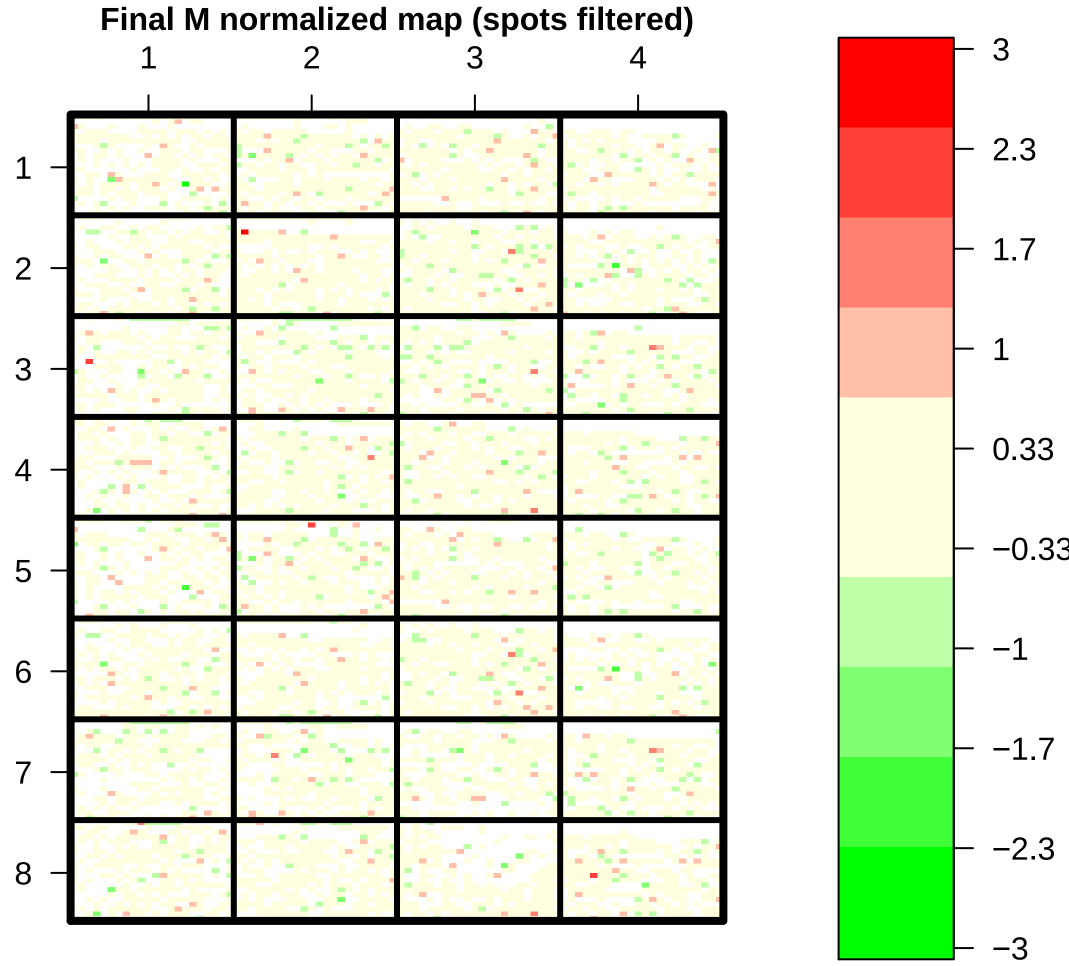
Final M normalized map (spots filtered)
1
2
3
4
1
2
3
4
5
6
7
8
3
2.3
1.7
1
0.33
−0.33
−1
−1.7
−2.3
−3

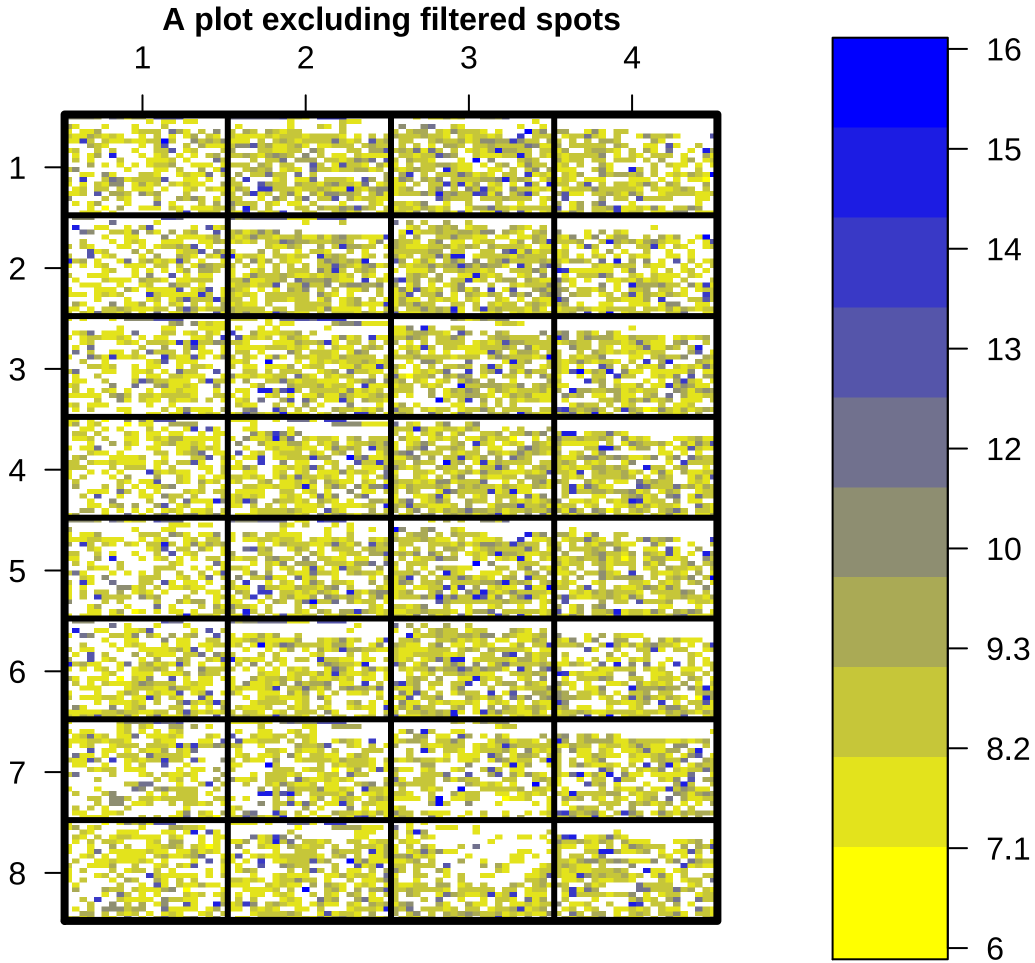

A plot excluding filtered spots
1
2
3
4
1
2
3
4
5
6
7
8
16
15
14
13
12
10
9.3
8.2
7.1
6
