# Supplementary figures and images for: Goulphar: rapid access and expertise for standard two-color microarray normalization methods
Source: BMC Bioinformatics. 2006 Oct 23;7:467. doi: 10.1186/1471-2105-7-467 (PMC1626094; doi:10.1186/1471-2105-7-467)

**Number of unfiltered spots in each block  
(alert if smaller than 200 )**

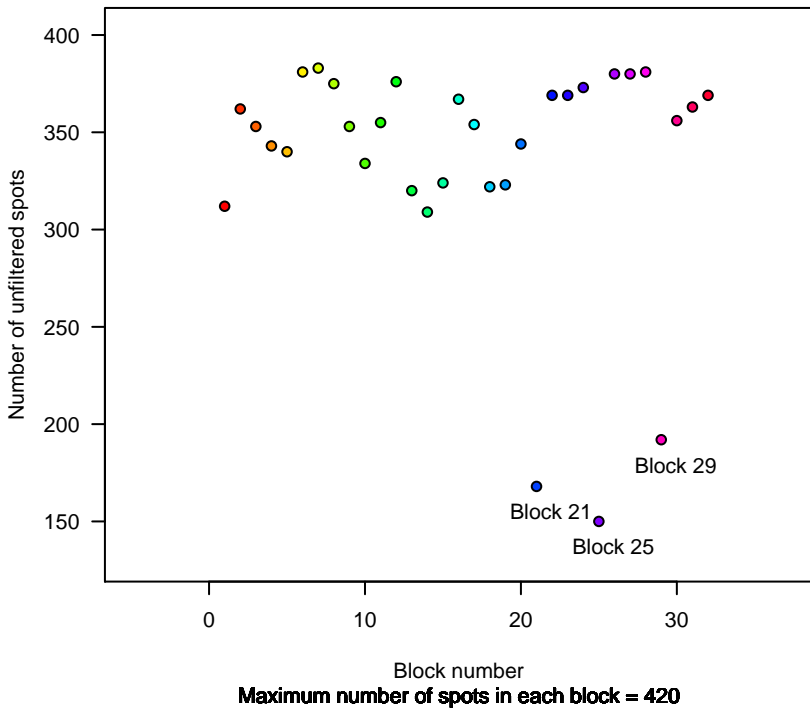

Supplement: Additional file 4 — Print-tip lowess quality control plot. This plot is obtained when the "print-tip group lowess" normalization method is selected. It displays the number of spots, in each print-tip group, kept for normalization once the filtration process has been performed (i.e. removing artifactual spots). The lowess correction method is sensitive to the number of spots used for the calculation, and this method is not appropriate if the there are too few spots in one block. Blocks with too few spots are displayed on the graph (here blocks 21, 25 and 29) to help the user to assess the efficiency of local lowess normalization. The maximum number of spots to be expected in each block, from the slide layout analysis, is displayed below the x-axis label. The experiment on which this plot is based was performed on yeast microarrays (Véronique Tanty personal communication). [file 1471-2105-7-467-S4.pdf]
