# Supplementary material for: Goulphar: rapid access and expertise for standard two-color microarray normalization methods
Source: BMC Bioinformatics. 2006 Oct 23;7:467. doi: 10.1186/1471-2105-7-467 (PMC1626094; doi:10.1186/1471-2105-7-467)
Supplement: Additional file 5 — Goulphar script documentation. The documentation of the Goulphar script deals with all the options available for parameter file creation and all the outputs that can be obtained. [file 1471-2105-7-467-S5.PDF]

 Transcriptome Platform

GOULPHAR

- Different normalization methods available
- The parameter file param_goulphar.dat
- The output plot files
  - MA-plots
  - Box-plots
  - Background plots
  - Filtered spot plot
  - Log2(ratio) plots
  - Average log2(intensity) plot
  - Plot of the number of unfiltered spots by block
  - Plots of the log2(intensity) density each channel ot the array
  - M density plots
- The normalized data file
- How to launch Goulphar in your R console
- List of the files created

Different normalization methods available

These methods are based on the hypothesis that most of the probes on your microarray are not differentially expressed (verified using the M distribution plot).

- **The global median method**: This is a linear regression of the log2(ratio)s against the median log2(intensity). This type of normalization does not take into account the intensity artefacts involved by the Cy3 (green) and Cy5 (red) dyes. At low intensity, the Cy5 dye is more intense than the Cy3, whereas the Cy3 dye is brighter in the high intensities. So that these artefacts can be taken this into account, this method was replaced by non linear methods.

- **The global lowess method**: Lowess means "Locally weighted scatter plot smoothing". It's a non linear regression of log2(ratio)s against the average log2(intensity). It is based on overlaping windows that slide from the beginning to the end of intensity range across the data. In these windows, It computes local linear regressions that are joined together to form a smooth curve. This normalization takes into account intensity artefacts.

- **The print-tip lowess method**: This method enables the correction of the spatial intensity artefacts involved by the print-tips of the robot during the probe spotting step (the print-tips are different). It works on the same principle as the glogal lowess method. The difference is that the overlaping windows for local linear regression computation are limited to a single print-tip group. Each print-tip group has its own normalization curve. This method seems better than the global one but implies to have enought spots in the print-tip groups, so that it can be valid (See the plot of the number of unfiltered spots by block to help monitoring this limitation).

- **The print-tip median method**: This method is a linear regression of each print-tip log2(ratio)s against its median log2(intensity). As for print-tip lowess method, this method enables also the correction of the spacial intensity artefacts involved by the print-tips. This method does not have limitation on the spot number by bloc (print-tip group).

- **The global lowess method followed by a print-tip median method**: This method associates global lowess and print-tip median method. Each print-tip log2(ratio) median is then centered on 0.

The parameter file "param_goulphar.dat"

Goulphar needs filtering and normalization parameters. These parameters are written in a text tabulated file and loaded in R. The file name is defined in Goulphar as param_goulphar.dat.

param_goulphar.dat example:

To ease comprehension, it was openned in a spreadsheet software:

	A	B	C	D	E	F	G	H	I	J	K	L	M
1	result.file	software	foreground	do.flagremoval	do.bgcorr	do.saturating	saturating	do.diameter	diameter	norma	alert.printtip	imagefile	gal.file
2	genepix01_84a.gpr	genepix	0	all	0	1	60000	0	0	lmb	200	2	0
3													

List of the parameters:

**result.file**	The name of the result file you want to use (ex : **genepix01_84a.gpr**)
**software**	**genepix** if your result file is a gpr file<br>**spot** if your result file is a spot file

**foreground**	**0** if you use the spot median intensity<br>**1** if you use the spot mean intensity
**do.flagremoval**	**all** to filter out flagged spots<br>**none** to keep flagged spots<br>**-75;-50** to filter out specific flags, flag values are separated by a semicolon. Here 'Absent' and 'Not Found' spots are filtered out (**only available for genepix files**)
**do.bgcorr**	**0** no background subtraction<br>**1** median background intensity subtraction<br>**2** mean background intensity subtraction
**do.saturating**	**1** to filter out the spots saturating in one channel (threshold included)<br>**0** to keep the saturating spots
**saturating**	The threshold value from which you consider the spot is saturating (ex : **60000**)
**do.diameter**	**1** to filter out the spots with a small diameter (threshold included)<br>**0** to keep the small spots
**diameter**	The threshold value from which you consider the spot diameter too small (ex : **60**)
**norma**	**p** to use print-tip lowess method<br>**l** to use global lowess method<br>**m** to use global median method<br>**lmb** to use global lowess method followed by print-tip median method<br>**mb** to use print-tip median method
**alert.printtip**	the threshold value from which you consider the number of spot by block too small to use the print-tip lowess normalization (default: **200**)
**imagefile**	**1** to have a single pdf file with graphical outputs<br>**2** to have separate png files<br>**3** to have separate jpeg files
**gal.file**	the name of the gal file used with Spot (**only needed for spot file input**)<br>**0** if the file is a genepix file

The output plot files

The spots filtered out depends on the options you chose in the parameter file. They can be small spots, saturating spots and flagged spots. They will be excluded from the normalization and from most of the outputs plots. They will remain included if you don't filter them. The spots that match the threshold values defined for saturating and small spots are included in the filter.

- ### MA-plots before and after data normalization

M is the log base 2 of the ratio : **M = log2(Cy5/Cy3)**

A is the average of the log base 2 of the intensities : **A = (log2(Cy3 intensity) + log2(Cy5 intensity)) / 2**

Print-tip lowess normalization ("p")

Each curve is a lowess curve for one bloc.

Example 1: with background subtraction

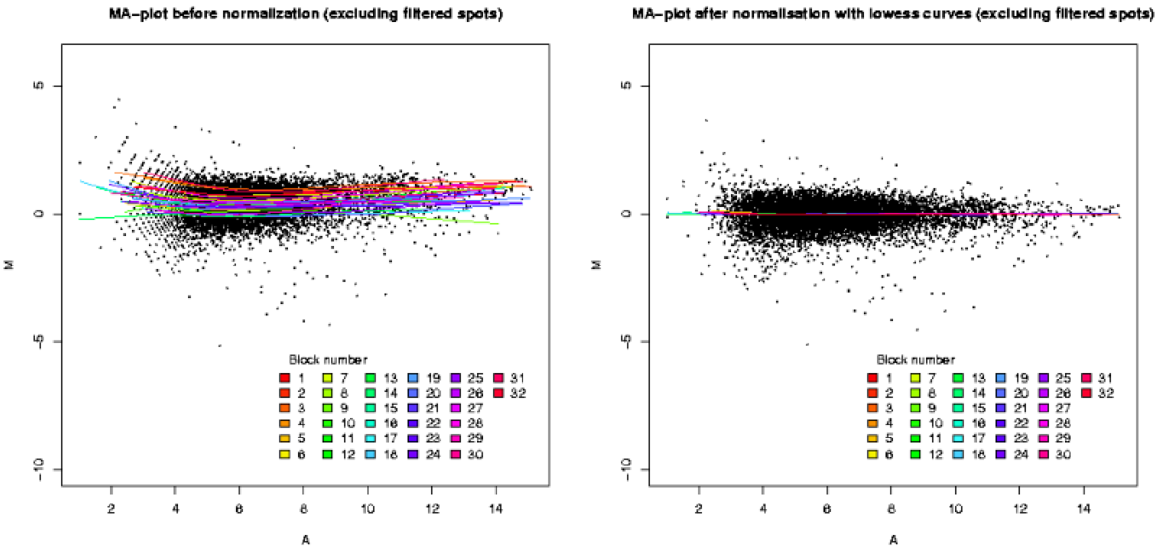

Example 2: without background subtraction

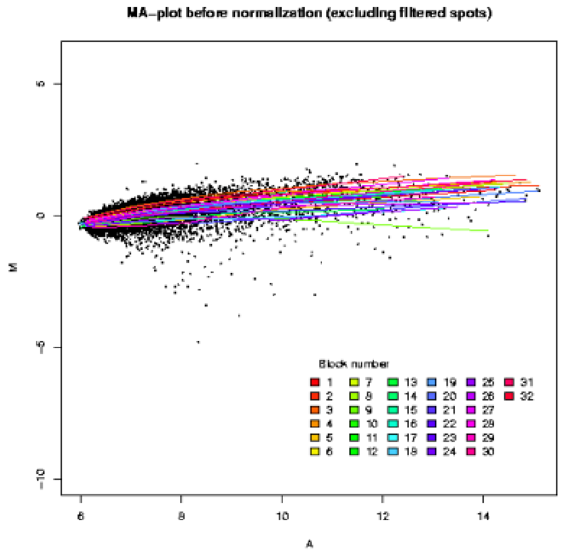

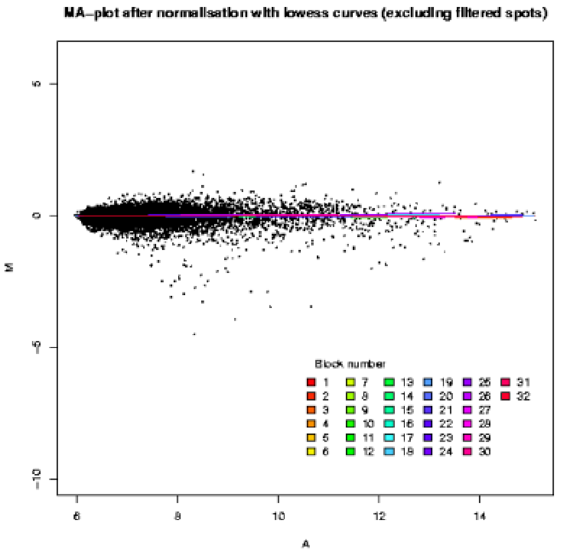

Global lowess normalization ("l")

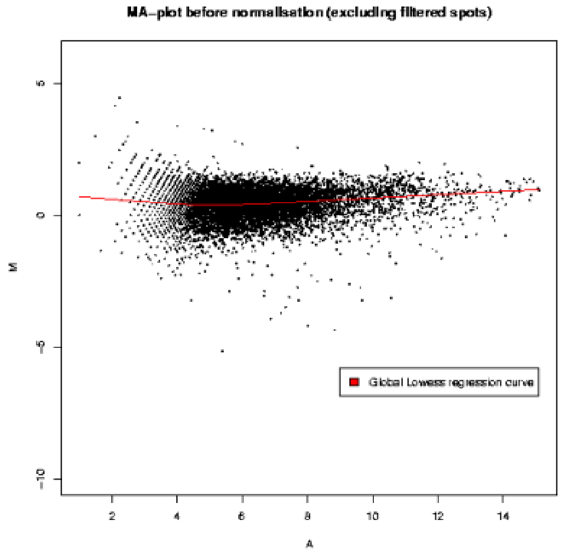

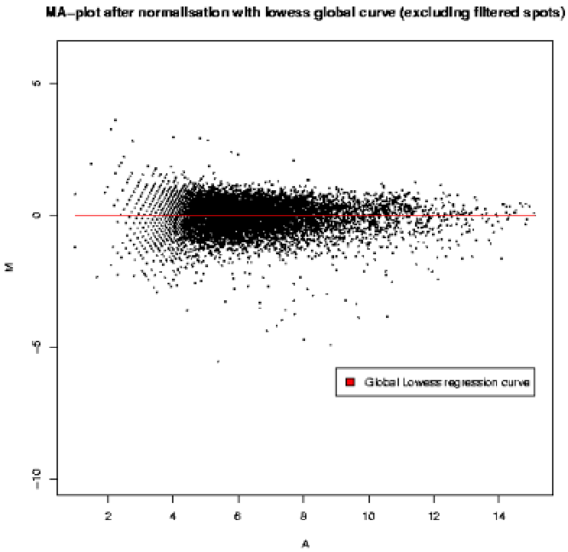

Global median normalization ("m")

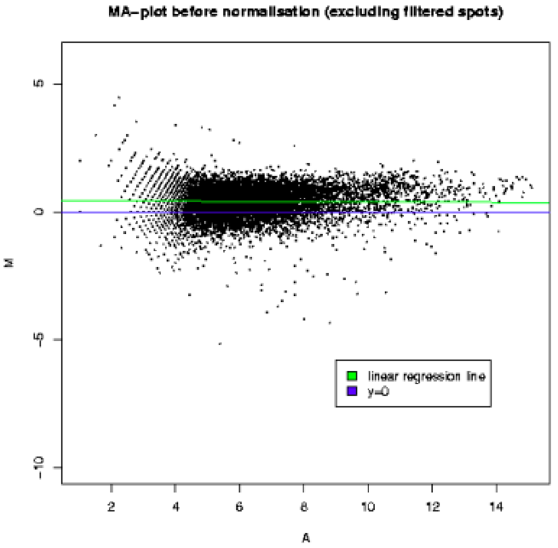

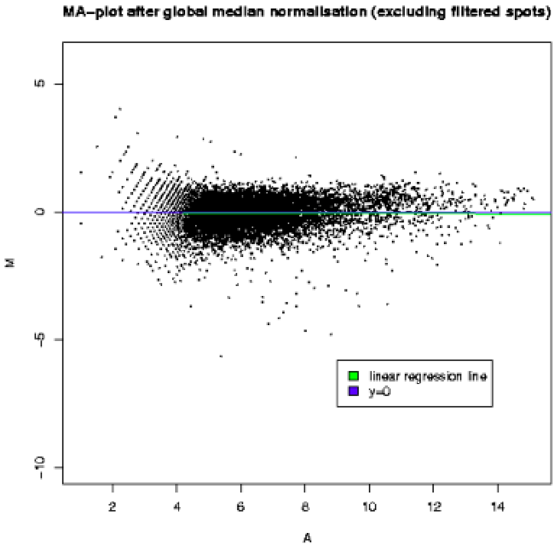

- **Box-plots before and after data normalization**

The box-plots allow the comparaison of the log2(ratio) distribution of all the print-tip groups of your microarray. These plots are generated for each normalization method so that you can have a picture of your data heterogeneity.

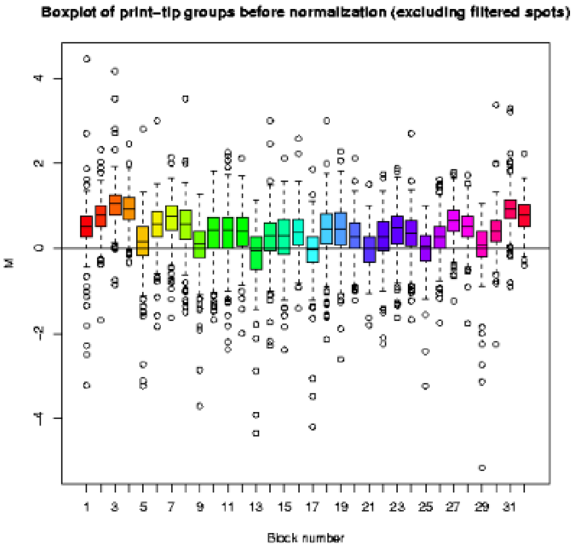

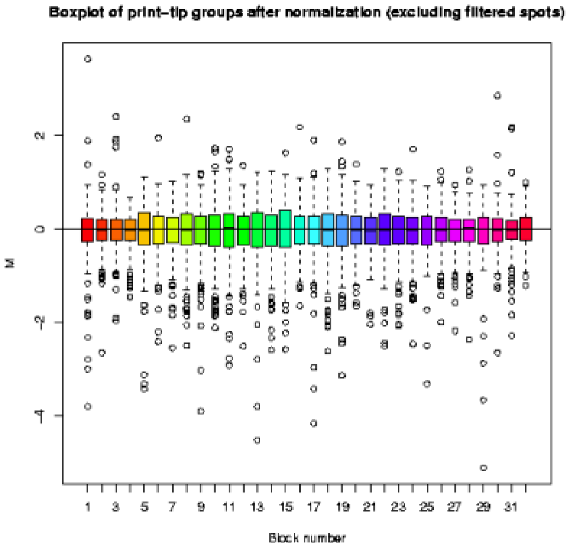

- **Background plots in each channel, with and without filtered spot (white spots)**

These plots are pictures of the background, in red (Cy5) and green (Cy3), on your array, first with all spots and then without filtered spots. Remember filtered spots can be the image analysis software flagged spots, small spots and saturating spots, depending on the options chosen before launching Goulphar.

Array map for the background representations

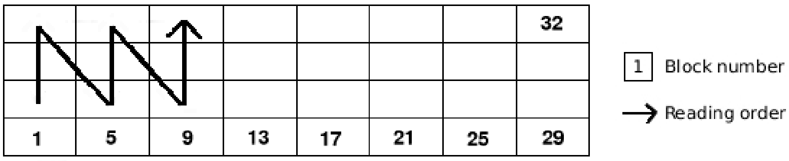

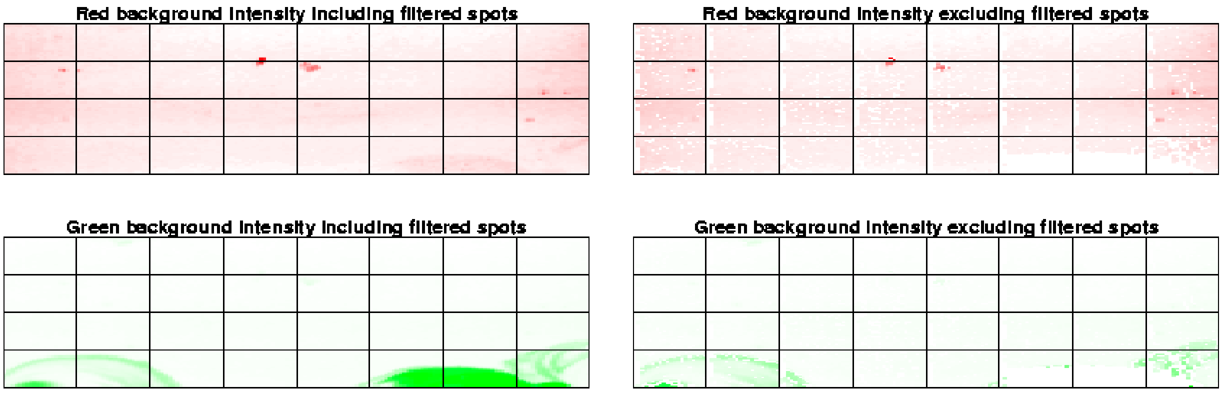

- **Filtered spot plot (yellow spots)**

Here is another filtered spot picture. Filtered spots can be, saturating spots (above the threshold selected), small diameter spots (below the threshold selected) and image analysis sofware flagged spots, depending on the filtering options you chose. If you don't activate any filter, the resulting plot is white. If you activate filters, the plot is white and yellow (Yellow spots are filtered spots).

Array map for the filtered spot representation

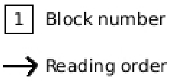

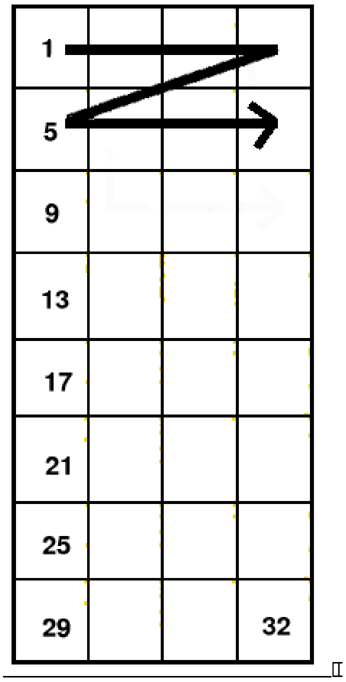

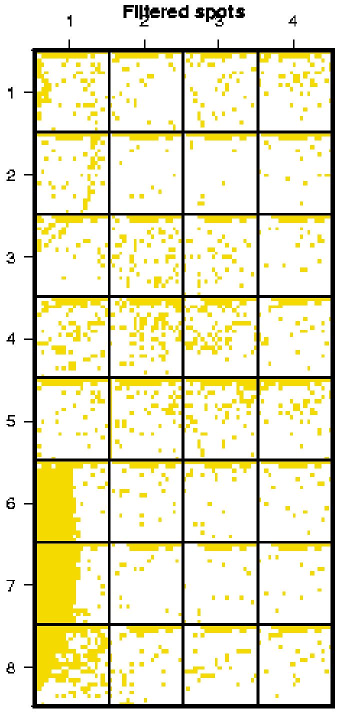

- **Log2(ratio) plots before and after normalization**

These plots enable you to trace local artefacts on the array: red or green area appear before normalization. These local artefacts should be corrected by the normalization step: after normalization, the log2(ratio) plot should be homogeneous. The scales are dynamic, be careful with these plots. When log2(ratio) is high, its color on the plot is red. When it's low, it's green and when it's close to 1, it's pale yellow.

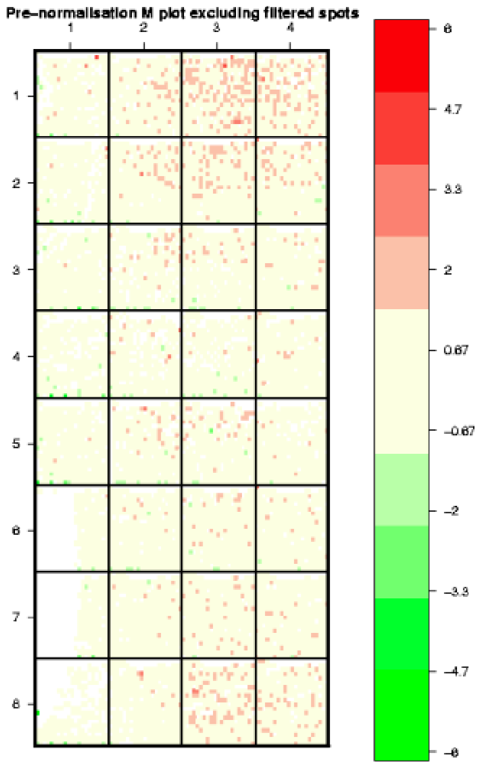

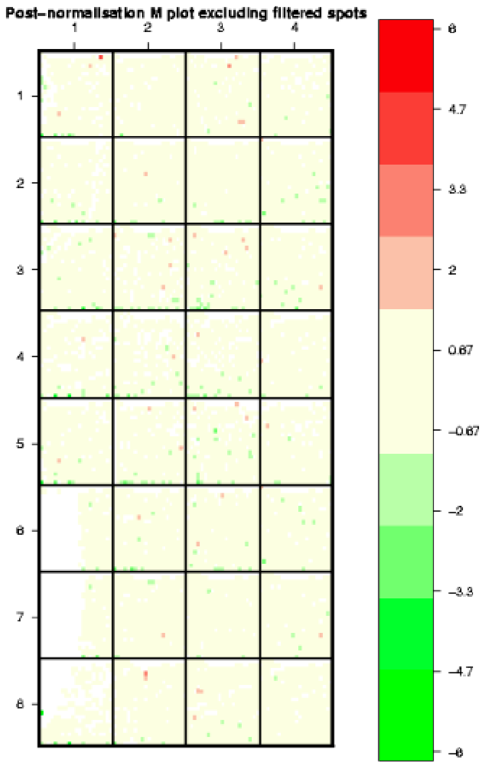

- **Average log2(intensity) plot**

This plot is a picture of the measured intensity: the more it's blue the higher the average intensity is. It allows you to be more confident with your log2(ratio). This plot also enable you to trace local artefacts. When average intensity is high, its color on the plot is blue. When

it's low, it's yellow.

Here, the average intensity is globaly weak:

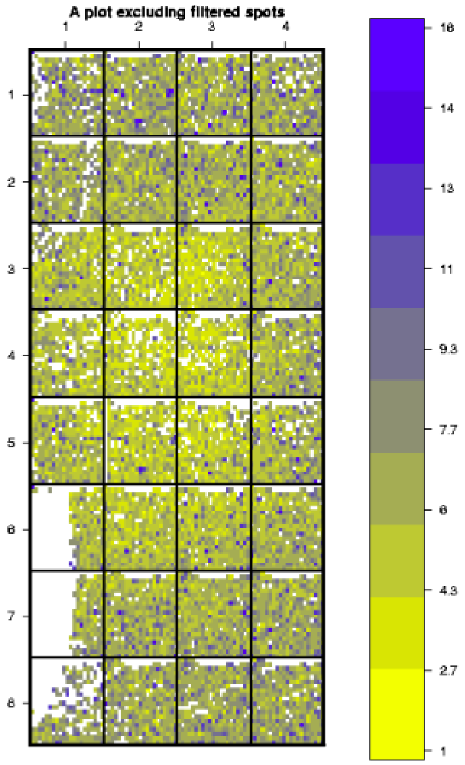

There, you can see a local artefact on each block:
the signal intensities of the upper part of each block are higher than the intensities of the lower part. This effect is due to a difference of concentration between the probe plates used.

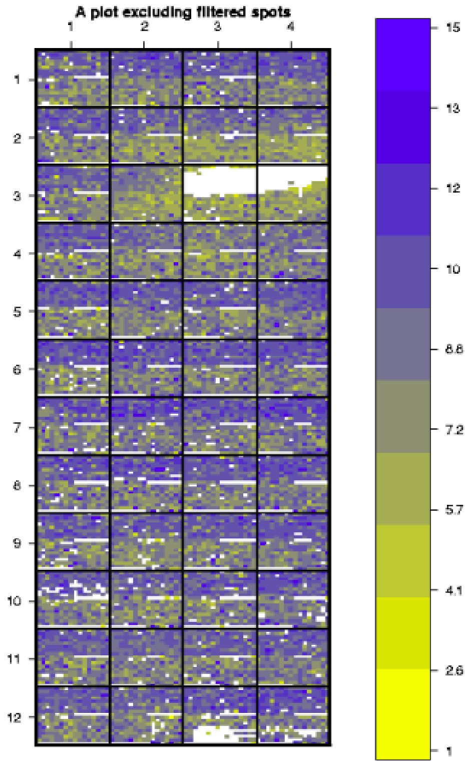

- **Plot of the number of unfiltered spots by block (only for print-tip lowess normalization)**

This plot enable the identification of blocks that have many spots filtered out (The block number appears when the spot number is lower than threshold fixed by the user, default value is 200). The theoretical spot number is mentionned under the x coordinate axe. In case the block spot numbers are too low, the print-tip lowess normalization might not be adapted. It might be better to use a global lowess based method.

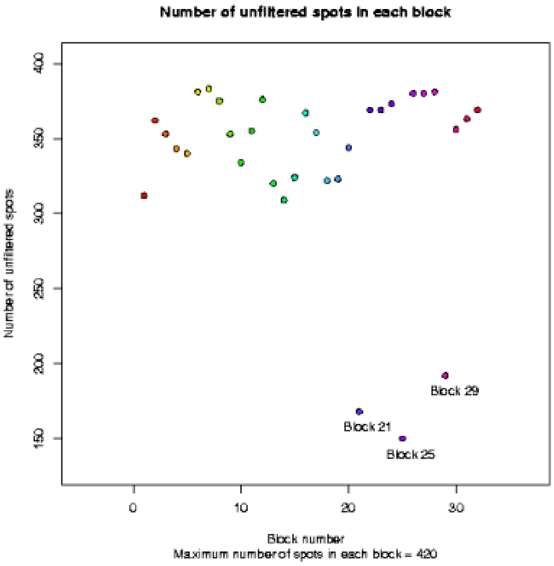

- **Plots of the log2(intensity) density in each channel ot the array**

These plots allow the user to check if the dye balance has been correted by the normalization method used.

Before normalization

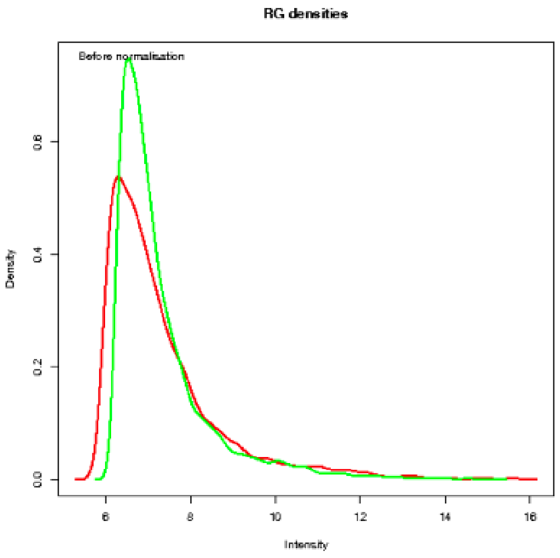

After normalization

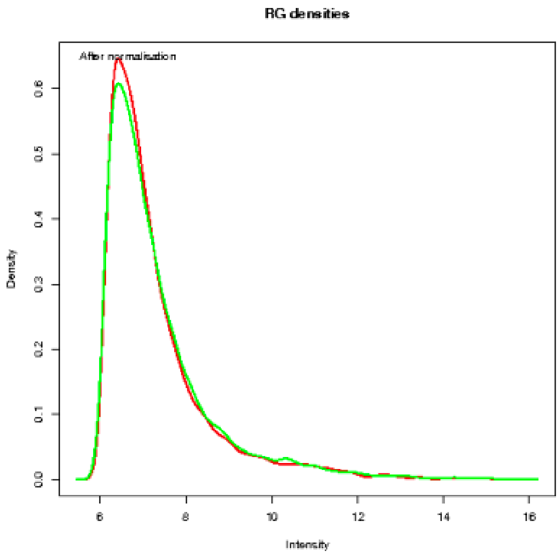

- **M density plots**

This plot allow the user to check the normality of the data before and after normalization.

Before normalization

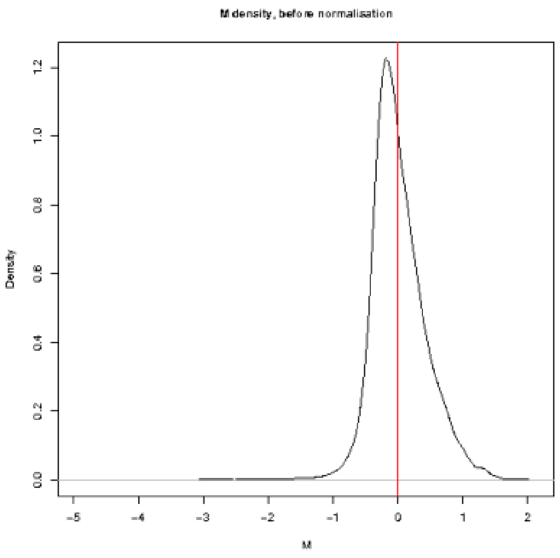

After normalization

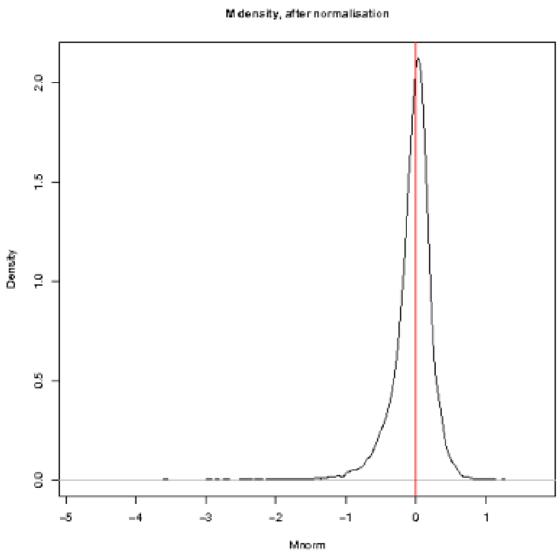

The normalized data file

A tabulated text file is generated at the end of the script. The script outputs are automatically saved in the current directory, that is to say the directory were is launch Goulphar (See "How to launch Goulphar"). The columns R, G, Rb and Gb are the raw foreground and background intensities, respectively for Cy5 (red) and Cy3 (green). None of these intensities are modified by the normalization step. It's the same concerning the average log2(intensity), the A column. The Mnorm column contains normalized data. The background intensity columns, Rb and Gb, don't exist if you chose to subtract background at the begining. The NA values are "not available values", resulting from empty spots called "Undef" or from filtering.

	A	B	C	D	E	F	G	H
1	ID	Name	R	Rb	G	Gb	Mnorm	A
2	RAT1	yor048c	NA	NA	NA	NA	NA	NA
3	unknown (15I24)	yor142w-b	NA	NA	NA	NA	NA	NA
4	DFR1	yor236w	101	56	101	81	5.628961e-01	4.906891
5	unknown (16A4)	yor311c	103	56	110	78	5.687615e-03	5.277294
6	RET3	ypl010w	299	55	213	76	3.205388e-01	7.514385
7	unknown (16I4)	ypl105c	137	56	140	76	-1.192080e-01	6.169925
8	unknown (16M4)	ypl200w	155	56	144	77	1.292156e-01	6.347723
9	unknown (16A8)	yor315w	96	56	118	85	-2.896730e-01	5.183161
10	unknown (16E8)	ypl014w	366	57	302	104	1.485658e-01	7.950410
11	unknown (16I8)	ypl109c	NA	NA	NA	NA	NA	NA

12	HRR25	ypl204w	559	57	580	118	-3.958506e-01	8.911646
13	HSH49	yor319w	118	57	148	82	-6.017362e-01	5.987566
14	CTF19	ypl018w	88	58	90	72	9.189657e-02	4.538408
15	unknown (16I12)	ypl113c	68	58	78	72	-2.969344e-02	2.953445
16	unknown (16M12)	ypl208w	323	57	266	71	-4.521859e-02	7.831306
17	PRO2	yor323c	417	56	319	71	5.276817e-02	8.225026
18	RAD1	ypl022w	116	55	125	71	-3.147032e-01	5.842812
19	IDI1	ypl117c	265	55	231	70	-1.284399e-01	7.522581
20	PUS1	ypl212c	189	55	222	70	-6.934175e-01	7.157008
21	SNC2	yor327c	378	54	247	70	3.805031e-01	7.903728
22	SKS1	ypl026c	141	55	148	70	-2.923391e-01	6.355833
23	TPT1	yol102c	170	57	464	313	-9.293990e-01	7.029292

How to launch Goulphar in your R console

- Launch R
- Go to the directory where the result file to be normalized and the parameter file param_goulphar.dat are.
  - Use the command line **setwd()** (ex:setwd("c:/Documents and Settings/Test")) or use "Change dir" in the menu bar.
  - The output files of Goulphar will be saved there.
  - Use **getwd()** to ensure you're in the right place if you're not sure.
- Type **source("/where_is_the_script/goulphar")**
- R loads the packages used in the script and executes the script
  Here is what you see if the script is running correctly:

```
> source("Goulphar.R")
Loading required package: Biobase
Loading required package: tools
Welcome to Bioconductor
         Vignettes contain introductory material.  To view,
         simply type: openVignette()
         For details on reading vignettes, see
         the openVignette help page.
[1] "parameter file OK"
[1] "GPR file = genepix01_84a.gpr"
[1] "Foreground = median foreground"
[1] "Remove flag = remove all flags"
[1] "Background subtraction = no background subtraction"
[1] "Saturating spot filter = saturating spot threshold = 60000"
[1] "Spot diameter filter = No spot diameter filter"
[1] "Normalisation type = global lowess followed by a block median normalisation"
[1] "Graphical output= in separate png files"
Read genepix01_84a.gpr
[1] "No Subtraction of local background"
[1] "Gbplot and Rbplot"
[1] "Rbplotflagged and Gplotflagged"
[1] "Fplot"
[1] "norma= global lowess followed by a block median normalisation"
[1] "ma plot"
[1] "manorm plot"
[1] "final manorm plot"
[1] "box plot"
[1] "boxnorm plot"
[1] "final boxnorm plot"
[1] "RG densities plot"
[1] "M densities plot"
[1] "MPlot"
[1] "MPlotnorm"
[1] "finalMPlotnorm"
[1] "APlot"
[1] "Result file done"
[1] "Finished"
> 
```

- Quit R using the command **q()**
- The plots and the normalized data file are in your current directory.

  If you want to run different normalization methods or test different parameters, be aware that Goulphar will overwrite the existing files. Don't forget to rename your different files !

List of the files created

The pdf and text output file names come from the gpr file name normalized by Goulphar (Your_gpr_file_name).

If you choose the single pdf graphical output:

**"Your gpr file name".pdf**	A pdf file containing all your plots
**"Your gpr file name"_norm.txt**	Your normalized data file

If you choose the separate png or jpeg graphical files:

**Gbplot**	Green background plot
**Gbplotflagged**	Green background plot, without filtered spots
**Rbplot**	Red background plot
**Rbplotflagged**	Red background plot, without filtered spots
**Fplot**	Filtered spot plot
**ma**	Before normalization MA plot
**manorm**	Post normalization MA plot or intermediate normalization MA plot in case you use a lowess global normalization followed by a print-tip median normalization
**finalmanorm**	final normalization MA plot, in case you use a lowess global normalization followed by a print-tip median normalization
**box**	Before normalization box plot
**boxnorm**	Post normalization box plot or intermediate normalization box plot in case you use the a lowess global normalization followed by a print-tip median normalization
**finalboxnorm**	final normalization box plot, in case you use a lowess global normalization followed by a print-tip median normalization
**valid_spot**	Plot of the unfiltered spot number in each block (only for the print-tip lowess normalization)
**RGdensities**	Before normalization density plot for both channel intensity
**RGdensities_norm**	Post normalization density plot for both channel intensity
**Mdensities**	Before normalization M density plot
**Mdensities_norm**	Post normalization M density plot
**Mplot**	Before normalization M log2(ratio) plot
**Mplotnorm**	Post normalization log2(ratio) plot, or intermediate log2(ratio) plot in case you use a lowess global normalization followed by a print-tip median normalization
**finalMplotnorm**	Final normalization log2(ratio) plot in case you use a lowess global followed by a print-tip median normalization
**Aplot**	Average log2(intensity) plot
**"Your gpr file name"_norm.txt**	Your normalized data file

If you want to run different normalization methods or test different parameters, be aware that Goulphar will overwrite the existing files. Don't forget to rename your different files !
